# A Retrospective Case Series Reporting the Safety of a Polyvinyl Alcohol (PVA) Composite Filler for Facial Augmentation

**DOI:** 10.1111/jocd.70823

**Published:** 2026-03-30

**Authors:** Wen‐ting Wang, Zu‐meng Ya, Cui‐ling Pu, Fang Yang, Wei Yang, Ya Tao, Chu Han, Xin‐meng Zhang, Shi‐wei Wang, Mu‐yan Zou, Li‐na Gou, Bao‐feng Ding, Xia Lou

**Affiliations:** ^1^ Department of Plastic Surgery Guangzhou Yestar Medical Aesthetic Hospital Guangzhou Guangdong China; ^2^ Chongqing Vcharm Plastic Surgery Hospital Chongqing China; ^3^ Department of Plastic Surgery Shenzhen Yestar Medical Aesthetic Hospital Shenzhen Guangdong China; ^4^ Beijing Huaxia Medical Beauty Hospital Beijing China; ^5^ Department of Plastic Surgery Wuhan Wuzhou Plastic Surgery Hospital Wuhan Hubei China; ^6^ Department of Medical Cosmetology Beijing Naimi Medical Aesthetics Clinic Co., Ltd Beijing China; ^7^ Department of Plastic Surgery Jinan Hans Plastic Surgery Hospital Jinan Shandong China; ^8^ Medical Department Imeik Technology Development Co., Ltd Beijing China; ^9^ Department of Medical Cosmetology Nanjing Bravou Medical Beauty Hospital Nanjing China

## Abstract

**Background:**

A novel polyvinyl alcohol (PVA)‐based filler has been developed for durable facial augmentation. Despite its clinical application, long‐term real‐world data regarding its safety profile remain limited.

**Objective:**

This study aimed to retrospectively assess the long‐term safety profile and clinical outcomes of this PVA‐based filler for facial augmentation.

**Methods:**

A multi‐center, retrospective case series was conducted across eight aesthetic clinics in China. Medical records of patients who received the PVA‐based filler for facial augmentation between January 2022 and January 2025 with a follow‐up of at least 18 months were reviewed. Adverse events (AEs) were extracted from the original medical charts and follow‐up logs and were classified by time of onset, type, and severity. A post hoc descriptive efficacy assessment using the Global Aesthetic Improvement Scale (GAIS) and FACE‐Q was performed on a sub‐cohort with complete photographic documentation exceeding 12 months.

**Results:**

A total of 279 patient charts were included, with a mean follow‐up period of 24.5 ± 7.2 months. Three patients (1.07%) reported at least one AE (totaling 4 events), all of which were early‐onset, transient, and mild injection site reactions that resolved spontaneously. No late‐onset adverse events were reported in this cohort. The descriptive efficacy analysis (*n* = 18) demonstrated sustained corrective effects, with a 72.2% improvement rate on GAIS and high patient satisfaction scores on FACE‐Q.

**Conclusion:**

In this retrospective cohort, the PVA‐based composite filler demonstrated a low incidence of adverse events, with absence of documented late‐onset complications in 279 patients. Descriptive observations of long‐term efficacy are encouraging; further prospective, controlled studies are warranted to objectively substantiate its durability and performance.

## 1 Introduction

Facial aging is a multifactorial process characterized by volume loss due to bone resorption and fat pad atrophy, as well as the cutaneous degradation of collagen and elastin [1, 2] In recent years, minimally invasive procedures have gained prominence for facial rejuvenation [3] Among these, hyaluronic acid (HA) fillers are the most widely used due to their favorable safety profile, biocompatibility, and reversibility with hyaluronidase [4] However, the principal limitation of HA fillers is their inherent biodegradability. Although cross‐linked HA substantially increases resistance to enzymatic degradation, its clinical efficacy is temporary, typically lasting 6 to 12 months, which necessitates periodic retreatments to maintain aesthetic correction [5].

Consequently, biostimulatory fillers, such as those containing calcium hydroxylapatite (CaHA) or poly‐L‐lactic acid (PLLA), have been developed for the long‐lasting aesthetic effects [6, 7] While these agents achieve durability by initiating a controlled foreign‐body inflammatory response that promotes neocollagenesis, this mechanism also carries an inherent risk of adverse events, such as nodule or granuloma formation [8, 9]. These clinical limitations highlight an unmet need for a dermal filler that combines long‐lasting results with a more favorable safety profile.

The novel PVA‐based composite filler ([Product name and manufacturer blinded for peer review]) is composed of polyvinyl alcohol (PVA) microspheres homogeneously suspended in a non‐crosslinked HA and hydroxypropyl methylcellulose (HPMC) gel. This formulation employs a dual mechanism of action. The HA‐HPMC gel provides immediate volumization upon injection, while the stable PVA microspheres facilitate long‐term tissue augmentation [10, 11].

Despite this promising mechanism and the established safety of its components, robust, long‐term clinical data evaluating the safety and efficacy of this specific PVA‐based composite filler in a real‐world setting remain limited. Therefore, this retrospective, multi‐center, post‐marketing study was designed to assess the long‐term safety and efficacy of this PVA‐based composite filler for facial augmentation.

## 2 Method

### 2.1 Study Design

This study followed a multicenter, retrospective case series design. Data were retrieved from the medical records of patients treated across eight aesthetic clinics in China between January 2022 and January 2025. This study was conducted in strict adherence to the principles of the Declaration of Helsinki. As this was a retrospective analysis of existing medical records where all data were de‐identified and anonymized, Institutional Review Board (IRB) approval was not required.

### 2.2 Data Collection and Case Selection Criteria

A standardized data extraction form was utilized to systematically retrieve anonymized data from original medical records. To mitigate the risks of underreporting and selection bias, data review and extraction were performed independently by two investigators. Any discrepancies were resolved through consensus discussion. Investigators reviewed clinical notes, procedure logs, and follow‐up records from January 2022 to January 2025 to identify and document all AEs associated with the PVA‐based filler.

The inclusion criteria were defined as patients aged ≥ 18 years who underwent facial augmentation with the PVA‐based filler and had a well‐documented follow‐up of at least 18 months after the final treatment session. Patients with a history of other fillers in the treatment area during the follow‐up period, or cases with incomplete medical records were excluded.

### 2.3 Safety Assessment

Data regarding adverse events (AEs) were extracted from medical records and systematically classified. Events were classified primarily based on the clinical manifestation and etiology, including injection site reactions, infections, hypersensitivity, implantation defects, skin discoloration, and vascular compromise [12].

All AEs were categorized into early and late reactions based on the time of onset. Consistent with established consensus, early adverse events were defined as those occurring within 4 weeks post‐injection [.12] These events primarily included common injection site reactions, such as erythema, edema, pain/tenderness, bruising, and itching, but also encompassed other acute complications such as bumps/lumps, asymmetries, or signs of vascular compromise.

Late adverse events were defined as those occurring beyond 4 weeks post‐injection, including delayed‐onset inflammatory nodules, foreign‐body granulomas, biofilm‐mediated complications, implant migration, systemic responses, persistent discoloration, and tissue necrosis.

Additionally, the severity of all AEs, any clinical management or interventions required, and the final outcome of each event were assessed and recorded.

### 2.4 Efficacy Assessment

A total of 18 patients were identified for a post hoc descriptive efficacy assessment based on the availability of concurrent efficacy data. Inclusion in this sub‐cohort required charts to meet the following criteria: [1] availability of complete, high‐quality, and standardized photographic documentation at baseline and at a minimum of 12 months post‐procedure; and [2] clear documentation of the initial treatment protocol, including specific injection site and injection volume of each injection site. Written informed consent was explicitly obtained from the patients for the reproduction and publication of the accompanying photographs.

Assessment of aesthetic improvement in the sub‐cohort was conducted by two independent, blinded investigators using the Global Aesthetic Improvement Scale (GAIS) by comparing baseline and 12‐month post‐treatment standardized photographs. Additionally, patient‐reported satisfaction was quantified using the FACE‐Q “Satisfaction with Facial Appearance” module, administered during a retrospective recall assessment to evaluate the patients' perception of their aesthetic outcomes.

### 2.5 Statistical Analysis

The sample size of the study cohort was defined as the cumulative number of all eligible patients treated during the study period. Descriptive statistics were utilized to summarize patient demographics and efficacy outcomes (GAIS and FACE‐Q). Continuous variables were expressed as mean ± standard deviation (SD), while categorical variables were presented as frequencies and percentages. The incidence rates for adverse events were calculated along with 95% confidence intervals (CIs) using the Wilson score method to assess precision. All statistical analyses were performed using SPSS Statistics Version 22.0 (IBM Corporation, Armonk, NY, USA).

## 3 Result

### 3.1 Participant Demographics and Baseline Characteristics

A total of 279 patient cases were retrieved and analyzed for this study. The study population was predominantly female (255, 91.4%) with 24 male patients (8.6%). The mean age of the participants at the time of the first treatment was 29.5 ± 13.1 years (range 22–65 years). The mean follow‐up period was 24.5 ± 7.2 months (range 18–36 months). A majority of patients (*n* = 198, 71.0%) had previous experience with other aesthetic treatments, most commonly HA fillers. None of the patients had any history of permanent filler injections or contraindications to the procedure.

The mean volume of PVA‐based filler administered per treatment session was 1.85 ± 0.6 mL (range 0.5–4.0 mL), and injections were performed across multiple facial regions, primarily including the nose, forehead, and jawline.

### 3.2 Safety Profile

The overall safety profile of the PVA‐based composite filler demonstrated a low rate of mild injection‐site reactions and an absence of documented late‐onset complications. Of the 279 patients included in the analysis, three (1.07%; 95% CI: 0.36%–3.11%) reported at least one AE during the observation period, accounting for 4 events. All documented AEs were classified as early‐onset, transient, and injection‐site related. Specifically, the reported events comprised two cases of mild edema/swelling, one case of mild bruising, and one case of mild tenderness at the injection site. These events resolved spontaneously without requiring pharmacological intervention, typically within 3–7 days post‐treatment. Intermittent cold compresses and gentle massage were recommended to these patients for symptomatic relief, and these management approaches effectively reduced swelling and discomfort, aligning with standard post‐procedure care.

No late‐onset adverse events were reported or documented in any of the 279 retrieved cases (0%; 95% CI: 0%–1.35%). Critically, among all retrieved cases, no documented cases of infection, hypersensitivity reactions, persistent skin discoloration, foreign‐body granulomas, biofilm, migration of materials, systemic responses, persistent discoloration, or thrombogenesis were observed. All safety findings are summarized in Table 1.

**TABLE 1:** Summary of Adverse Events (*N* = 279 Patients).

Adverse Events	Onsite Time	Patients (n)	Events (n)	Incidence (%)	Severity	Outcome (Management)
Early Onset AEs	≤ 4 weeks	3	4	1.07%	Mild	Recovered
Edema/Swelling		1	2	0.36%	Mild	Recovered; (Cold compress applied.)
Bruising/Hemorrhage		1	1	0.36%	Mild	Recovered
Pain/Tenderness		1	1	0.36%	Mild	Recovered
Early Onset AEs	> 4 weeks	0	0	0	/	/

### 3.3 Efficacy Assessment

A total of 18 eligible cases were included for a descriptive efficacy assessment. These patients presented with complete, high‐quality, and standardized photographic documentation spanning a follow‐up period of at least 12 months, alongside clear treatment records. This exclusively female sub‐cohort had a mean age of 31.4 ± 10.6 years, with the forehead (66.7%) serving as the primary treatment areas.

**FIGURE 1:**
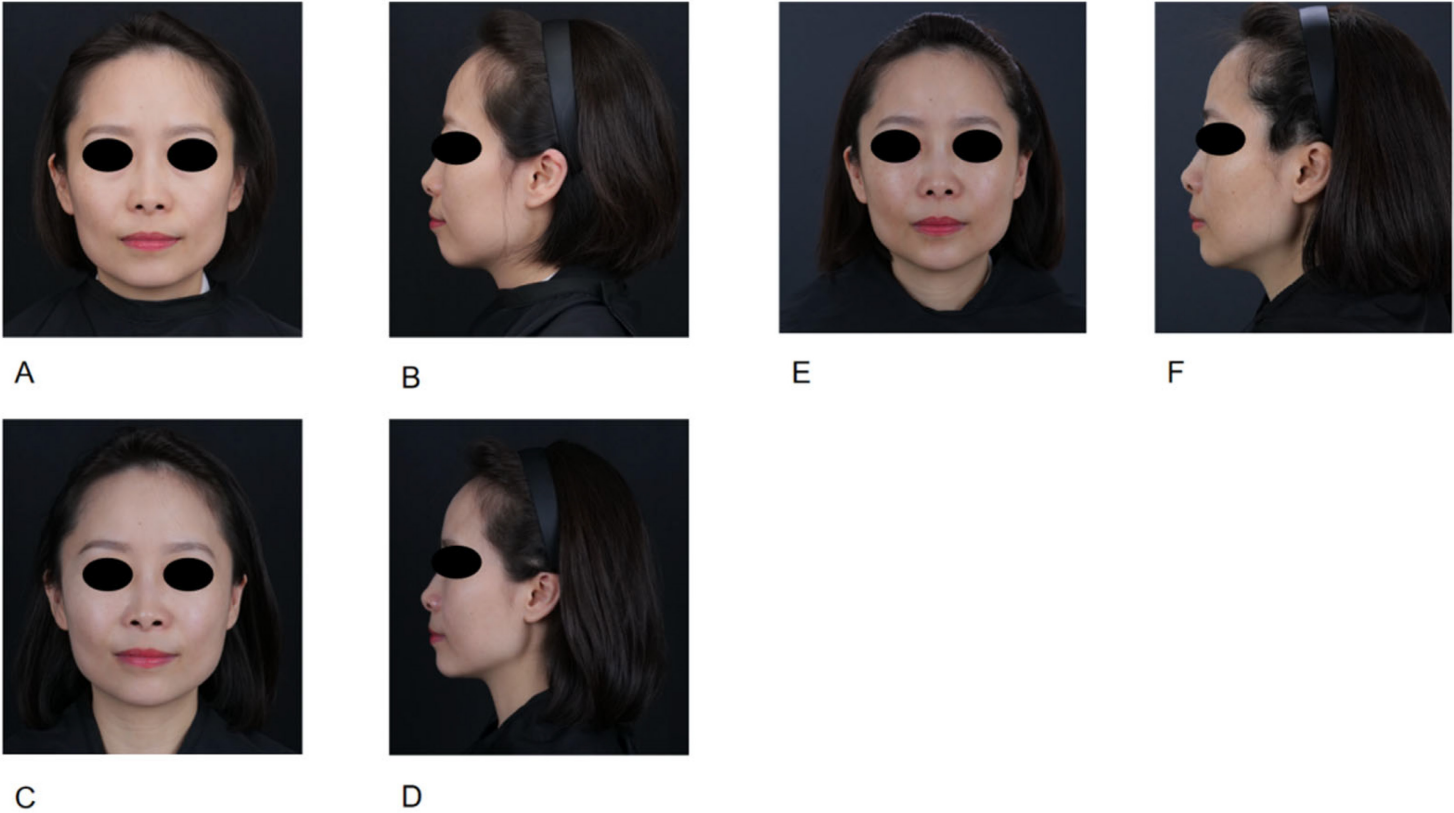
Clinical outcomes of a female treated with PVA‐based filler using 2 mL for the mandibular margin, 1 mL for the forehead, and 1 mL for the temporal region, demonstrating long‐term facial contouring efficacy. (A, B) Pre‐treatment frontal and lateral views. (C, D) Frontal and lateral views at 6 months post‐treatment. (E, F) Frontal and lateral views at 12 months post‐treatment.

**FIGURE 2:**
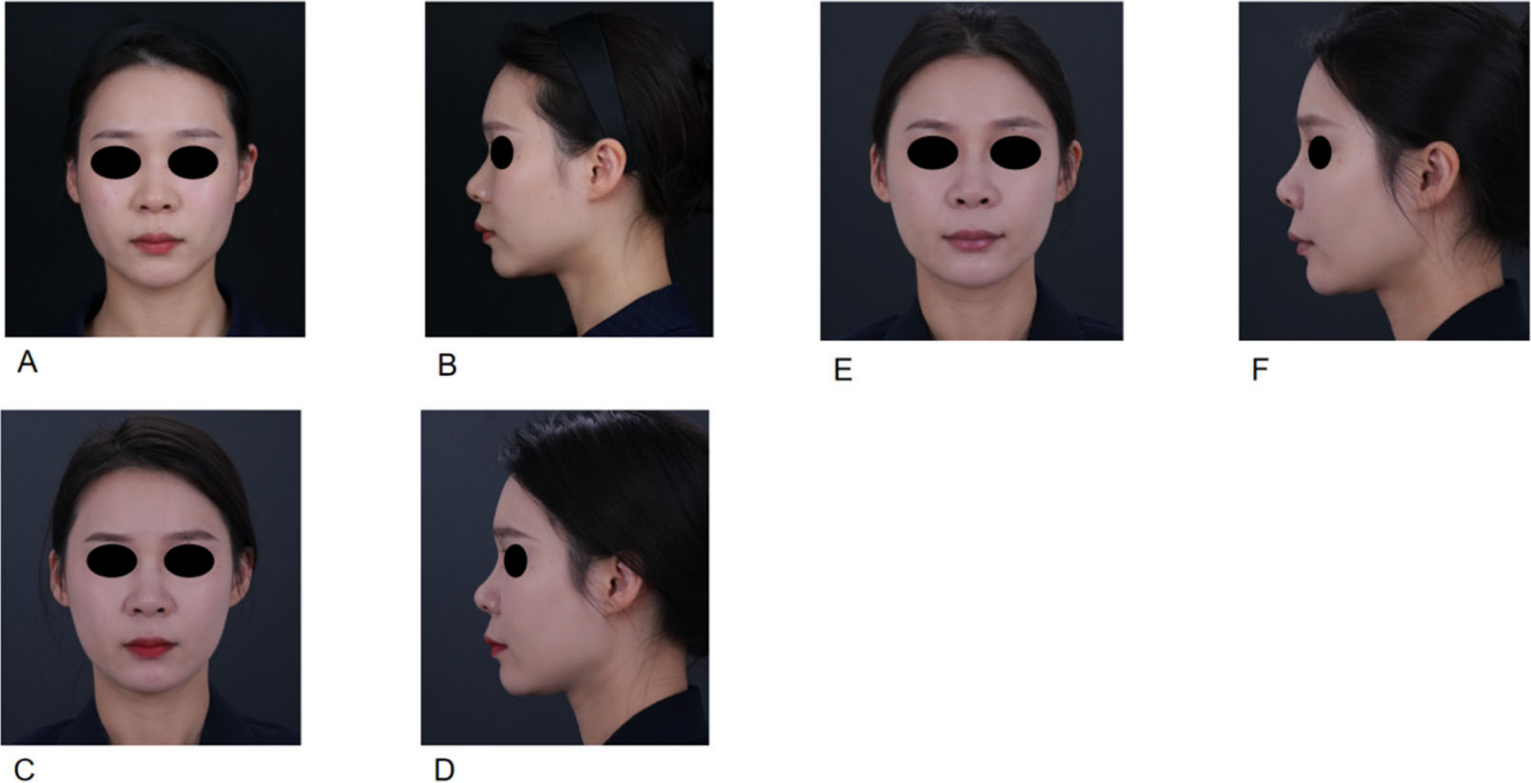
Clinical outcomes of a female treated with PVA‐based filler (1.5 mL for the supraorbital ridge, 1 mL for the lateral orbital region, 0.5 mL for the pretarsal roll, and 0.6 mL for the nasal radix) demonstrating precision augmentation efficacy in the periorbital and nasal regions. (A, B) Pre‐treatment frontal and lateral views. (C, D) Frontal and lateral views at 6 months post‐treatment. (E, F) Frontal and lateral views at 12 months post‐treatment.

The GAIS improvement rate at 12 months indicated that the majority of patients (72.2%) were graded as improvement, with 16.7% rated as “very much improved,” 33.3% rated as “much improved” and 22.2% as “improved,” with a mean score of 2.28 ± 1.32. These objective findings were aligned with the patient‐reported outcomes. The mean FACE‐Q Satisfaction with Facial Appearance score at the 12‐month follow‐up was 78.56, reflecting a high level of sustained patient satisfaction with the treatment results. The following two cases illustrate the sustained effects and high patient satisfaction observed over a period exceeding 12 months (Figures 1, 2). Serial photography documents the progression from baseline (A, B) to 6 months post‐treatment (C, D) and 1 year post‐treatment (E, F).

Case 1 (Figure 1) demonstrates the efficacy of the PVA‐based filler in facial contouring. This patient received a total of 4 mL of PVA‐based filler, with 2 mL along the mandibular margin, 1 mL in the forehead, and 1 mL in the temporal region. At the 6 month follow‐up (C, D), comparisons with baseline photographs (A, B) revealed significant improvement in facial convexity and jawline definition. Notably, these aesthetic enhancements were sustained at the 1 year follow‐up (E, F). The mandibular contour remained sharp and defined, and the volume in the forehead and temporal areas showed no visible degradation, indicating structural stability of the PVA‐based filler over the 12 month period.

Case 2 (Figure 2) indicates the efficacy of the PVA‐based filler in volume enhancement. The treatment involved 1.5 mL in the brow arch, 1 mL in the lateral orbital region, 0.5 mL for pretarsal roll augmentation and 0.6 mL in the nasal radix. The 6 month assessment (C, D) demonstrated an improvement in the periorbital region and nasal projection compared to pre‐treatment (A, B). At 1 year post‐treatment (E, F), the results remained stable and natural, suggesting that the filler maintains its shape and position well in high‐mobility and thin‐skinned areas without migration or significant resorption.

## 4 Discussion

This multicenter, retrospective case series, comprising 279 patients with an average follow‐up of 24.5 months, provides real‐world evidence supporting the clinical safety of the PVA‐based composite filler ([Product name and manufacturer blinded for peer review]) for facial augmentation. The overall incidence of AEs was 1.07% (3/279 patients). Reported AEs were limited to early‐onset, transient, and mild injection‐site reactions, including edema, bruising, and tenderness, all of which resolved without pharmacological intervention. Notably, no late‐onset adverse events were documented in this patient group during the follow‐up period.

While the inherent methodological heterogeneity necessitates caution when comparing incidence rates from this retrospective real‐world study directly with those from prospective randomized controlled trials, the observed low incidence of early‐onset AEs and the complete absence of delayed complications remain clinically significant findings relative to other filler materials. Systematic reviews generally report a higher frequency of immediate injection‐site reactions following HA injection compared to our findings, with rates of approximately 40.7% for swelling and 10.8% for bruising [13] alongside a rare but existing risk of delayed inflammatory reactions, ranging from 0.02% to 0.5% [14] PLLA and CaHA fillers are well‐documented to carry a risk of nodule and granuloma formation, which is associated with their inflammatory mechanism of action. Although advancements in microsphere morphology and formulation optimization have been introduced to enhance the safety profile of these fillers, previous studies have demonstrated late‐onset incidence rates ranging from 0.9% to 8.6% for PLLA and approximately 0.85% to 3% for CaHA [15, 16] The absence of any late‐onset AEs in our cohort over an average of 24.5 months suggests a stable augmentation profile with a reduced risk of both the delayed immunogenicity and the chronic inflammatory responses associated with biostimulation.

The observed safety profile is likely attributable to the unique properties of the PVA microspheres. PVA is a well‐established biomaterial with a long history of use in medical applications [17] including ophthalmology products [18] and drug delivery vehicle [19] Furthermore, its high mechanical strength, low friction coefficient, and favorable biocompatibility have made it an ideal candidate for implantation materials such as artificial articular cartilage [20].

The use of PVA microspheres for soft tissue implantation introduces specific physical and biological properties that are critical for determining the long‐term tissue response and mitigating the risk of complications [21] Preclinical studies have demonstrated that the PVA microspheres elicit minimal inflammatory responses, confirmed by both H&E staining and low expression of the inflammatory marker IL‐6, and remained stable throughout the entire 12‐month observation period [22].

The absence of late‐onset AEs in our clinical cohort is consistent with the non‐inflammatory tissue response documented in preclinical models, where PVA microspheres elicited minimal macrophage activation. While histological evaluation was not conducted in this retrospective clinical analysis, these preclinical data provide a plausible biological basis for the material's long‐term tolerability.

Furthermore, the particle size of the microspheres is strictly controlled within the 20–45 μm range, effectively avoiding phagocytosis by macrophages, while simultaneously ensuring injectability and a natural aesthetic result [23, 24] In addition to these physical dimensions, the hydrophilic nature of PVA confers favorable biocompatibility to the microspheres [25] This hydrophilicity creates a protective hydration layer that effectively inhibits the adsorption of proteins [26] Consequently, this hydrophilic nature likely attenuates the inflammatory response and reduces the risk of granuloma formation.

In terms of efficacy, descriptive analysis of the sub‐cohort indicates sustained aesthetic improvement accompanied by high patient satisfaction. This durability is likely attributed to the unique composition of the filler. While the HA‐HPMC carrier undergoes gradual metabolism, the PVA microspheres serve as a sustained physical scaffold for tissue support.

Despite these positive findings, this study has several limitations. First, and most critically, the absence of documented severe adverse events (SAEs) in this cohort does not equate to a definitive absence of risk. Given the retrospective design, there is an inherent potential for under‐detection due to surveillance bias. Specifically, complications managed at outside institutions or those that did not prompt a return visit may not have been captured in the analyzed medical records. Second, although no late‐onset AEs were identified, the sample size of 279 patients may be insufficient to detect extremely rare complications. Furthermore, while a 2‐year follow‐up is substantial, longer surveillance is required to definitively assess the safety of materials over the long term. Finally, efficacy assessments were based on a post hoc analysis of available data, which may introduce potential bias. Therefore, future prospective, controlled studies are necessary to objectively substantiate these results.

In conclusion, this multicenter retrospective case series of 279 patients indicates a low incidence of adverse events associated with the PVA‐based composite filler in a real‐world clinical setting. The observations suggest a safety profile characterized primarily by transient injection‐site reactions and an absence of documented late‐onset complications in this cohort.

## Author Contributions

Wen‐ting Wang, Zu‐meng Ya, Cui‐ling Pu, Fang Yang, Wei Yang, Ya Tao, Chu Han, and Xin‐meng Zhang performed the research (clinical data acquisition). Shi‐wei Wang, Mu‐yan Zou, and Xia Lou designed the study. Hui Song and Bao‐feng Ding contributed essential reagents or tools. Shi‐wei Wang, Mu‐yan Zou, and Xia Lou analyzed the data. Mu‐yan Zou and Shi‐wei Wang wrote the paper. Each author has participated sufficiently in the work to take public responsibility for appropriate portions of the content and agreed to be accountable for all aspects of the work. All authors have read and approved the final manuscript.

## Ethics Statement

The study was compliant with the ethical requirements of the Declaration of Helsinki.

## Consent

The patients depicted in the figures have provided written informed consent for the publication of their photographs.

## Conflicts of Interest

The authors declare no conflicts of interest.

## Data Availability

The data that support the findings of this study are available from the corresponding author upon reasonable request.
